# Urine SERPINC1/ORM1 as biomarkers for early detection of lupus nephritis in MRL-lpr mice

**DOI:** 10.3389/fimmu.2023.1148574

**Published:** 2023-09-08

**Authors:** Young-Eun Kim, Eun-Ju Lee, Kyunggon Kim, Do Hoon Kim, Mi Ryeong Jeong, Jiyoung Yu, Seokchan Hong, Chang-Keun Lee, Bin Yoo, Yong-Gil Kim

**Affiliations:** ^1^ Department of Rheumatology, Asan Medical Center, University of Ulsan College of Medicine, Seoul, Republic of Korea; ^2^ Convergence Medicine Research Center, Asan Institute for Life Science, Asan Medical Center, Seoul, Republic of Korea; ^3^ Department of Biomedical Sciences, University of Ulsan College of Medicine, Seoul, Republic of Korea

**Keywords:** lupus nephritis, systemic lupus erythematosus, biomarker, proteomics, serpin peptidase inhibitor clade C (antithrombin) member 1 (SERPINC1), alpha-1-acid glycoprotein (ORM1), interleukins

## Abstract

**Background:**

To evaluate the usefulness of urine SERPINC1 and ORM1 as biomarkers for early detection of lupus nephritis (LN).

**Methods:**

Using proteomics, we screened for potential urine biomarkers that differentiate LN from systemic lupus erythematosus (SLE) patients without nephritis. In addition, urine levels of target biomarkers were measured by ELISA in 13- and 23-week-old MRL-lpr (murine model for LN) and MRL/MpJ mice. Histological analysis was also performed on the kidneys of 23-week-old mice.

**Results:**

Urine SERPINC1 and ORM1 were elevated in SLE patients with newly diagnosed LN compared with SLE patients without LN (SERPINC1, AUC=.892, P<.001; ORM1, AUC=.886, P<.001). Levels of urine SERPINC1 and ORM1 were also significantly higher in MRL-lpr mice than in MRL/MpJ mice at 13 and 23 weeks (SERPINC1: p<.01 and p<.001 at 13 and 23 weeks, respectively; ORM1: p<.01 at 13 and 23 weeks). In contrast, a significant difference in urine albumin between the two groups was only observed at 23 weeks (p<.001) not at 13 weeks (p=.83). Regarding the kidney pathology of MPL-lpr mice, urine ORM1 and urine albumin, but not urine SERPINC1, were positively correlated with the activity index (ORM1, rho =.879, p<.001; albumin, rho =.807, p=.003) and chronicity index (ORM1, rho =.947, p<.001; albumin, rho =.869, p<.001).

**Conclusion:**

We propose that urine SERPINC1 and ORM1 are novel biomarkers for early LN.

## Introduction

Lupus nephritis (LN) is one of the serious consequences of systemic lupus erythematosus (SLE), a leading cause of morbidity and mortality (1). Despite improvements in the treatment of LN, the prognosis remains poor if proper treatment is not provided in the early stages of the disease (2–4). Thus, there is a need for non-invasive biomarkers for early detection of disease. Traditional disease activity markers such as anti-dsDNA and complement are unsatisfactory for detecting early LN, and estimating the progress of treatment (5, 6). In addition, kidney biopsy, the gold standard for diagnosis of LN, poses risks of complications (7). Furthermore, since the presence of proteinuria or active urine sediment does not necessarily reflect the severity of the histologic features of LN (8), there is a need for biomarkers that are closely correlated with the histologic features of LN.

We have proposed Serpin peptidase inhibitor clade C (antithrombin) member 1 (SERPINC1) and alpha-1-acid glycoprotein (ORM1) as urine biomarkers for LN based on data obtained with the SWATH LC–MS liquid chromatography platform, a mass spectrometry-based proteomics analysis system (9). In the previous study, urine levels of ORM1 and SERPINC1 were elevated in newly diagnosed LN patients compared with healthy controls (HC) and systemic lupus erythematosus (SLE) patients without nephritis. SERPINC1, a member of the serpin C family, is known to regulate the blood coagulation cascade and anti-inflammatory activity (10). Levels of SERPINC1 are elevated in acute kidney disease, suggesting an anti-inflammatory action (11). ORM1, a member of the acute phase protein family, activates monocytes, induces T-cell proliferation, and promotes the secretion of proinflammatory cytokines (12).

Although SERPINC1 and ORM1 have been detected in newly diagnosed LN, their biological roles in early LN are unclear. We aimed to evaluate their utility as biomarkers for early LN in MRL/lpr mice, a murine model of LN (13).

## Materials and methods

### Mass spectrometry analysis for quantitative proteomics

We first sought potential urine biomarkers for LN in humans by quantitative proteomics. Urine samples from HC and patients with SLE at Asan Medical Center, Seoul, Korea, were collected between January 2019 and August 2020. All patients with SLE met the 2012 Systemic Lupus International Collaborating Clinics classification criteria for SLE (14). The patients with SLE were classified into three groups: (i) SLE patients without nephritis (isolated microscopic haematuria and pyuria were not considered nephritis), (ii) initial LN patients (iLN): SLE patients who were newly diagnosed with LN at the time of urine sample collection, and (iii) LN patients: SLE patients previously diagnosed with LN. We gathered information on age, sex, disease duration of SLE at the moment of urine collection. The laboratory data at the time of urine collection, including immunological parameters, such as anti-double-stranded DNA antibody (anti-dsDNA) and complement levels was also collected. Disease activity analysis was conducted utilizing the acquired systemic lupus erythematosus disease activity index-2000 (SLEDAI-2K) and non-renal SLEDAI-2K scores. The Institutional Review Board of Asan Medical Center approved the study (IRB No. 2013-0405). Written informed consent was obtained from all patients.

Methods for urine sample preparation and proteomic analysis have been described (15). In brief, 1 ml of each urine samples was dried using a CentriVap benchtop vacuum concentrator (Labconco, Cat No: # 7810010) and reconstituted in 500ul of 5% sodium dodecyl sulfate buffer with 50mM triethylammonium bicarbonate (pH 8.5). After assaying bicinchoninic acid, peptization was performed using an S-trap mini (Profiti, USA) according to the manufacturer’s instruction except for the trypsin/LysC setting (1:25 of protease: protein). An in-house urinary proteome library was generated using a pooled sample of peptides from all the groups, as described (15). Individual urine sample were analyzed by SWATH LC-MS using quadrupole time of flight mass spectrometry (TripleTOF^®^ 5600+ System, Sciex, USA) coupled with microflow LC (NanoLC 425, Sciex, USA) with pre-set parameters. The resulting spectrum data were processed using DIA-NN (version 1.7.10) with the in-house urine proteome spectral library to obtain relative quantitative information and identification. Each protein abundance was normalized using urine creatine amount of each patient at first then transformed to log2 value. After width adjustment, missing value replacement was performed. Receiver operating characteristics (ROC) analysis with normalized abundance of proteome was conducted using MedCalc (MedCalc Software Ltd, version 20.115) and additional ontology analysis and protein-interaction analysis using STRING (https://string-db.org/) was performed using the differentially expression proteins (DEPs) which were belonged to cluster 4 with MCL clustering option. In details, minimum required interaction score was set as default (medium confident 0.400) and inflation parameter was set to be 4 to identify the clusters.

### Mice

Female MRL/MpJ (#000486) (n=12) and MRL/MpJ-*Fas^lpr^* (#000485) (n=11) mice, aged 6-wk, were obtained from the Jackson Laboratories (ME, USA). The clinical activities of the mice were estimated via their levels of anti-dsDNA every two weeks to 18 weeks and at 21 weeks. Urine was collected from each mouse at 13 weeks and at 23 weeks and the level of urine protein/creatinine ratio (UPCR) of each mouse was performed at that time. At 13 and 23 weeks, the mice were sacrificed, and kidney tissue was obtained. All animal experiments were performed in accordance with the guidelines for animal care of the Animal Experimentation Committee of Asan Institute for Life S ciences (IRB No. 2020-14-099).

### Enzyme-linked immunosorbent assays

Serum anti-dsDNA antibodies were measured with a Mouse Anti-dsDNA antibody total IgG ELISA kit (Cat. 5110, Alpha Diagnostic, TX, USA). Levels of ORM1(abx575963, Abbexa, TX, USA), SERPINC1(ab108800, abcam, Cambridge, UK), albumin (ab108792, abcam, Cambridge, UK) and creatinine (ADI-907-030A, Enzo, NY, USA) in the urine of mice at 13 and 23-wk age were determined with commercially available ELISA kits, and adjusted by creatinine concentrations.

### Histologic analysis

Kidneys were fixed in 10% formalin for 24 hours at 4°C and embedded in paraffin. Standard protocols were used for hematoxylin and eosin (H&E), and periodic acid–Schiff (PAS) staining. Pathologic lesions of the kidney were evaluated blind by an animal pathologist, based on National Institutes of Health (NIH) activity and chronicity indices (16).

### Immunofluorescence staining of kidney tissue

Using the Opal method (Perkin Elmer), two primary antibodies were applied sequentially to a single slide. After deparaffinization in xylene and rehydration in ethanol, antigen was retrieved by microwave treatment in citrate buffer (pH 6.0). Primary rabbit antibodies for ORM1 (LSBio LifeSpan BioSciences, Inc, LS-C806250, 1:200) were incubated for 1 h in a humidified chamber at room temperature (RT), followed by detection using Polymer HRP Ms + Rb. Visualization of ORM1 was accomplished using fluorescein opal 520 (1:100), after which the slide was placed in citrate buffer (pH 6.0) and heated by microwave. The slides were then incubated with primary rabbit antibody for SERPINC1 (LSBio LifeSpan BioSciences, Inc, LS-B6624, 1:100) for 1 h in a humidified chamber at RT, followed by detection using Polymer HRP Ms + Rb. SERPINC1 was visualized using opal 690 (1:100). Finally, the slides were again placed in citrate buffer (pH 6.0) and heated by microwave. Nuclei were visualized with 4’,6-diamidino-2-phenylindole (DAPI) (1:500) and the sections were mounted to coverslips with mounting medium (Enzo) and scanned with a SLIDEVIEW VS200 and OlyVIA (Olympus, Germany). Representative area of SERPINC1 and ORM1 in tissue were measured by Olyvia software.

### Statistical analysis

All analysis was performed using GraphPad Prism 8.4.3 software (GraphPad Software, San Diego, CA, USA). Mann–Whitney U tests were performed for two-group comparisons. P values less than 0.05 were considered statistically significant. Significant differences are indicated with asterisks as follows: *p ≤ 0.05, **p ≤ 0.01, ***p ≤ 0.001, and ****p ≤ 0.0001.

## Results

### Proteomic analysis of LN biomarker candidates

Initially, biomarker candidates in urine samples from 25 HC and 79 SLE (SLE without nephritis; 37, iLN; 19, LN; 23) patients were validated. Of the SLE patients, 62 (78.5%) were women. The median ages of each group were 51.0 years (IQR 37.5-56.3), 43.0 (IQR, 23.0-49.5), 39.0 (IQR, 22.0-50.0) for SLE without nephritis, iLN, and LN patients, respectively, and the corresponding levels of proteinuria were 92.2 (IQR, 64.8-175.0) mg/g, 696.2 (IQR, 101.4-1511.9) mg/g, and 970.9 (IQR, 585.6-2328.1) mg/g. Three of 17 (17.6%) patients in the iLN group had proteinuria less than 500 mg/g. Further clinical characteristics of SLE patients were summarized in **Table 1**.

**Table 1:** Baseline characteristics of SLE patients.

	SLE without nephritis (N = 37)	iLN (N = 19)	LN (N = 23)	P value^a^
Age, years, median (IQR)	51.0 (37.5–56.3)	43.0 (23.0-49.5)	39.0 (22.0–50.0)	**0.005**
Female sex, n (%)	31 (83.7)	14 (73.7)	17 (73.9)	>0.999
Disease duration, months, median (IQR)	33.3 (18.0–64.2)	37.6 (0.8-88.8)	29.8 (8.1–63.6)	0.427
Serum creatinine, mg/dl, median (IQR)	0.60 (0.54–0.77)	0.73 (0.63-0.98)	0.67 (0.55–0.87)	0.348
UPCR, mg/g, median (IQR)	92.2 (64.8–175.0)	696.2 (101.4-1511.9)	970.9 (585.6-2328.1)	**<0.001**
Urine RBC ≥5/HPF, n (%)	2 (9.5)	4 (21.1)	8 (20.5)	0.470
Urine WBC ≥5/HPF, n (%)	2 (9.5)	4 (21.1)	9 (23.1)	0.299
C3, mg/dl, median (IQR)	98.1 (78.7–113.5)	63.9 (45.7)	79.1 (53.3–94.7)	**0.006**
C4, mg/dl, median (IQR)	21.1 (13.9–25.6)	7.9 (6.2-18.2)	16.2 (7.8–22.0)	**0.012**
Anti-dsDNA Ab, IU/ml, median (IQR)	8.2 (5.3–21.1)	124 (5.2-327.0)	14.9 (5.9–154.0)	**0.003**
ESR, mm/h, median (IQR)	20.0 (12.0–34.5)	24.0 (16.0-31.0)	21.0 (13.0–30.0)	0.762
CRP, mg/dl, median (IQR)	0.15 (0.10–0.26)	0.18 (0.10-0.45)	0.10 (0.10–0.37)	0.250
SLEDAI-2K, median (IQR)	6.0 (2.0–8.0)	8.0 (6.0-14.0)	8.0 (5.0–12.0)	0.260
Non-renal SLEDAI-2K, median (IQR)	4.0 (2.0–8.0)	4.0 (2.0–8.0)	4.0 (2.0–8.0)	0.838

a variables were compared by one-way analysis of variance (ANOVA).

SLE, systemic lupus erythematosus; LN, lupus nephritis; Cr, creatinine; UPCR, urine protein/creatinine ratio; RBC, red blood cell; HPF, high power field; WBC, white blood cell; Anti-dsDNA Ab, anti-double stranded DNA antibody; C3, complement protein 3; ESR, erythrocyte sedimentation rate; CRP, C-reactive protein; SLEDAI-2K, systemic lupus erythematosus disease activity index-2000. Bold values denote statistical significance at the P < 0.05 level.

**Figure 1:**
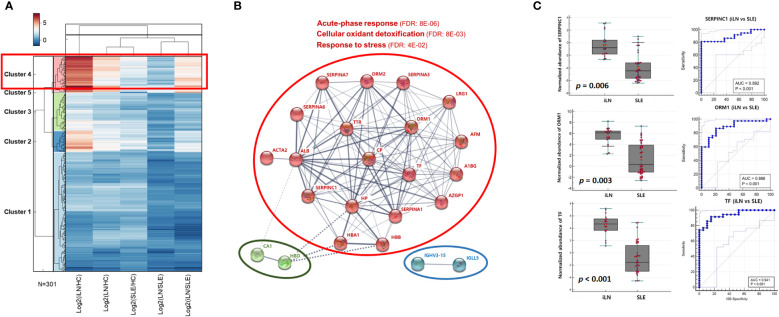
Comparative urinary proteome analysis **(A)** Hierarchical clustering analysis of the expression of proteins in the iLN group compared with the HC, SLE, and LN **(B)** Protein interaction map was generated using DEPs which increase more than 2 times with *p* < 0.05 in iLN group compared SLE group (Cluster 4) and gene ontology was performed using STRING embedded option. Red circle indicate that 19 protein-consisted network including SERPINA1 and ORM1 are belonged to 3 biological processes (acute-phase response, cellular oxidant detoxification and response to stress) **(C)** Box plots and ROC curves for SERPINC1 (upper panel) and ORM1 (lower panel) in the iLN group and SLE group; HC, healthy controls; LN, lupus nephritis groups; iLN, initial LN; SLE, systemic lupus erythematosus; DEPs, differentially expressed proteins; ROC; receiver operating characteristics.

From a quantitative proteome analysis of the 4 groups of urine samples using SWTAH LC-MS, we selected proteins which increased in the iLN group compared to the HC and SLE groups, and we performed a hierarchical clustering analysis of 5 combination (iLN/HC, LN/HC, SLE/HC, LN/SLE and iLN/SLE) using fold changes of the quantified urinary proteins. Of the resulting 4 clusters, cluster 4 showed protein expression patterns that increased in the iLN group compared to the HC and SLE groups (**Figure 1A**). Among 23 proteins which increased in iLN group than SLE group, 19 proteins including ORM1 and SERPINC1 showed a protein interaction network related to acute phase response (FDR 8E-06), cellular oxidant detoxification (FDR 8E-03) and response to stress (FDR 4E-02) (**Figure 1B**). Details of the clustering from STRING were prepared as **Supplementary Table 1**. In a comparative analysis of the iLN and SLE groups, SERPINC1 and ORM1 were more highly expressed in the iLN than the SLE group with p-values of 0.006 and 0.003 for SERPINC1 and ORM1, respectively. Areas under the curve (AUCs) of the ROC for SERPINC1 and ORM1 between the iLN group and the SLE group were 0.892 and 0.886, respectively (**Figure 1C**).

### Urine SERPINC1/ORM1 expression in MRL/lpr and MRL/MpJ mice

**Figure 2:**
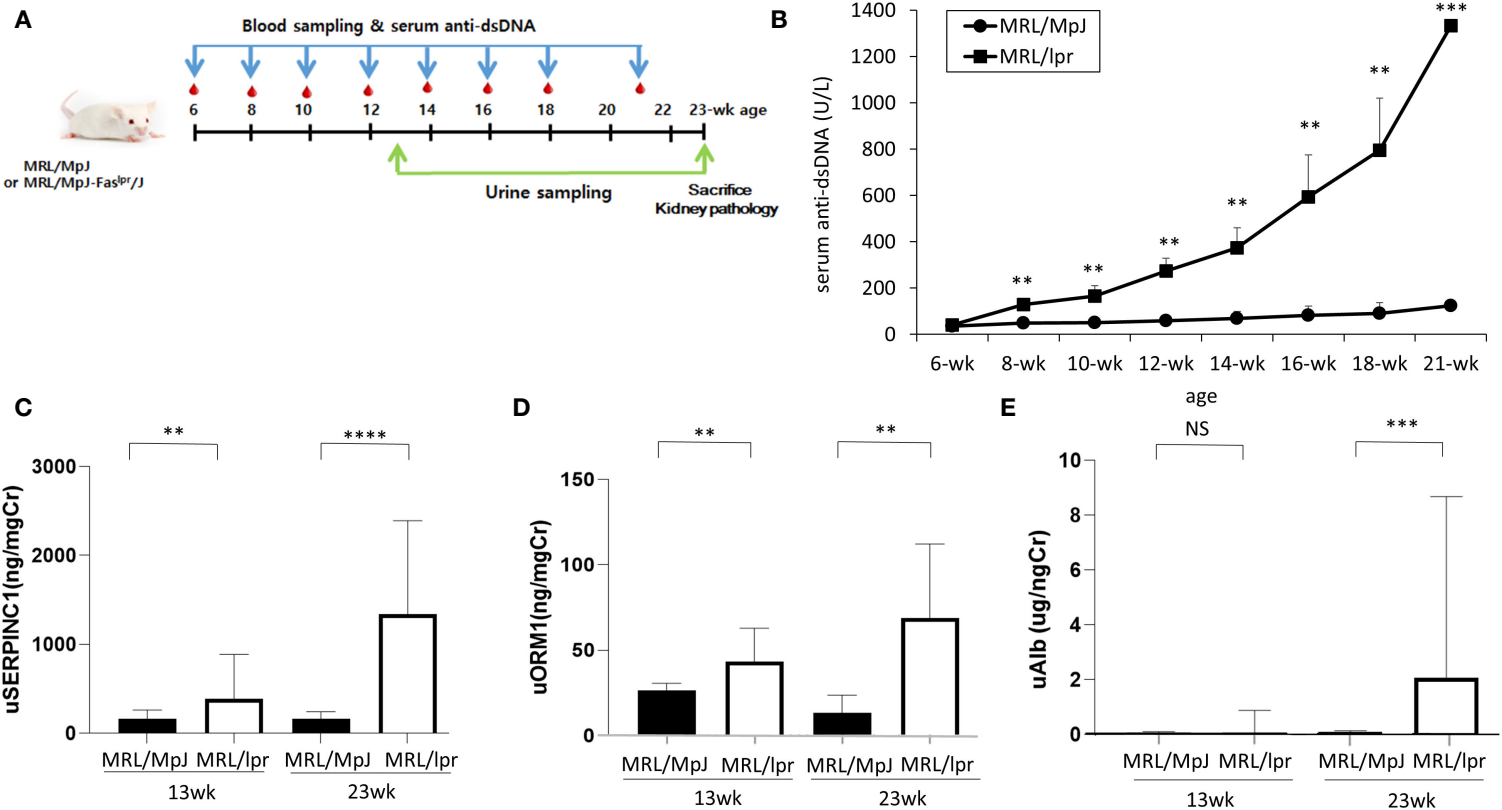
Serum and urine proteins in in MRL/lpr and MRL/MpJ mice. **(A)** Experimental design **(B)** Serum anti-dsDNA levels **(C)** urine SERPINC1, **(D)** urine ORM1, **(E)** urine albumin levels at 13 and 23 weeks, ** p<0.01, *** p<0.001.

A total of twenty-three MRL/lpr (n=12) and MRL/MpJ (n=11) mice were analyzed according to the experimental schedule (**Figure 2A**). Serum anti-dsDNA increased significantly more with time in the MRL-lpr than in the MRL-MpJ mice (**Figure 2B**).

To examine the usefulness of SERPINC1 and ORM1 for early detection of lupus nephritis, SERPINC1 and ORM1 urine levels were analyzed at 13 weeks and 23 weeks. SERPINC1 and ORM1 levels detected by ELISA were significantly higher in the MRL-lpr mice than the MRL/MpJ mice at 13 and 23 weeks (SERPINC1: p<.01 and p<.001 at 13 and 23 weeks, respectively; ORM1: p<.01 at 13 and 23 weeks) (**Figures 2C, D**). In contrast, we only detected a significant difference in urine albumin between the two groups at 23 weeks (p<.001) (**Figure 2E**).

### Histologic analysis of MRL/lpr mice

**Figure 3:**
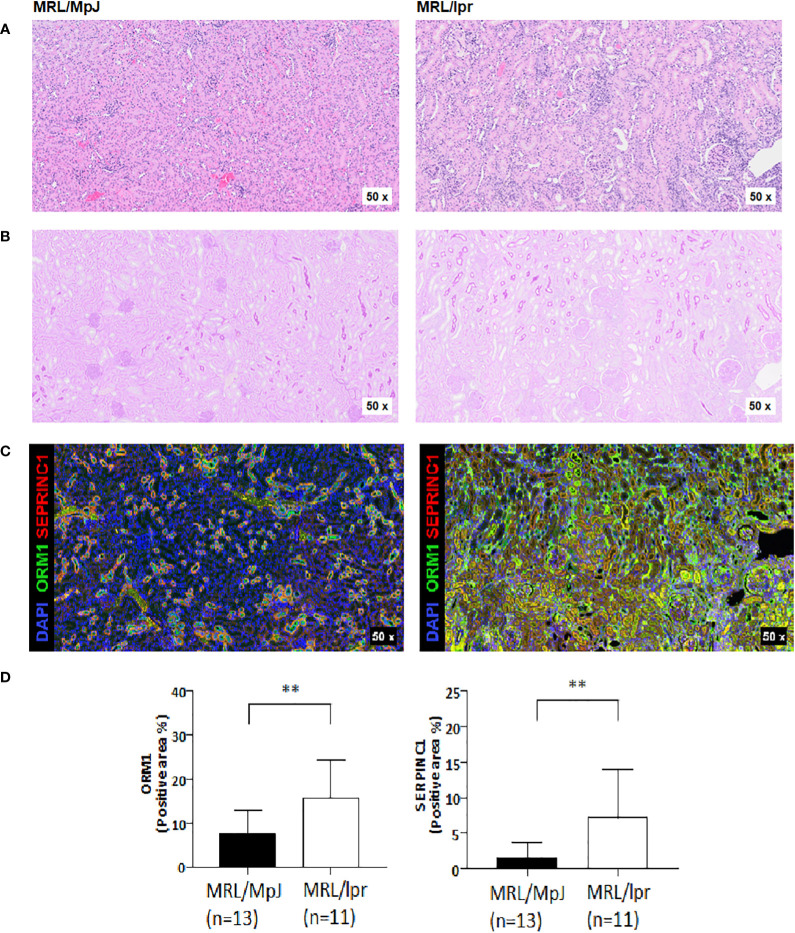
Histologic analysis of kidney tissues in MRL/lpr and MRL/MpJ mice at 23-week. **(A)** H&E-stained kidney tissue **(B)** PAS-stained kidney tissue **(C)** Opal-multiplexed immunofluorescence-stained kidney tissue **(D)** Quantification of positive areas, ** p<0.01.

Next, we performed histology analysis of MRL/lpr mice. Interstitial inflammation and endocapillary hypercellularity, which are indicators of activity index, were observed in all kidney tissues of MRL-lpr mice at 23 weeks. Interstitial fibrosis and global glomerulosclerosis, indicators of chronic index, were also observed in kidney tissues (**Figures 3A, B**). Activity index and chronicity index in MRL-lpr mice were median 8.0 (6.5-9.0), 2.0 (1.5-2.5), respectively. When we examined the expression of SERPINC1 and ORM1 in kidney tissue, SERPINC1 and ORM1 were primarily seen in the tubular membranes of immunofluorescence-stained kidney tissue (**Figure 3C**). The expression of SERPINC1 and ORM1 in kidney tissue was significantly higher in MRL/lpr mice than MRL/MpJ mice (**Figure 3D**). As shown in **Table 2**, urine ORM1 was found to be correlated with the activity index (rho =.879, p<.001) and chronicity index (rho =.947, p<.001) in the kidney tissue of MPL-lpr mice. The correlation with the histopathology index was stronger than for urine albumin (rho =.807, p=.003, for the activity index, and rho =.869, p=<.001 for the chronicity index). By the 23-week mark, notable ORM1/SERPINC1 expression was observed, aligned with histological findings indicative of LN. Intriguingly, even at the 13-week time point, ORM1/SERPINC1 expression was detected (statistical significance was achieved in ORM1, p <0.05) (**Supplementary Figure 1**).

**Table 2:** Correlation between potential urine biomarkers for LN and pathologic activity/chronicity indexes.

	Activity index			Chronicity index		
	Rho (95% CI)	R2	P value	Rho (95% CI)	R2	P value
Urine albumin	0.807 (0.410, 0.948)	**0.651**	**0.003**	0.869 (0.561, 0.965)	**0.755**	**<0.001**
Urine SERPINC1	0.082 (-0.545, 0.650)	0.007	0.811	0.022 (-0.587, 0.619)	0.000	0.949
Urine ORM1	0.879 (0.590, 0.968)	**0.772**	**<0.001**	0.947 (0.804, 0.987)	**0.897**	**<0.001**

Bold values denote statistical significance at the P < 0.05 level.

## Discussion

In this study we showed that urine SERPINC1 and ORM1 could be detected earlier in the LN murine model than urine albumin. In addition, urine ORM1 level, like urine albumin, was correlated with degree of activity and chronicity index. Therefore, we propose that urine SERPINC1 and ORM1 are novel biomarkers for early detection of LN.

Traditionally, biomarkers have been selected on the basis of the LN-associated pathophysiological pathways. However this is a biased approach that limits the detection of novel biomarkers (17, 18). In contrast, profiling proteins by proteomic analysis is an unbiased way to identify biomarkers such as ceruloplasmin and transferrin (19–21).

Recently, the development of techniques for high-throughput proteomic analysis has greatly advanced biomarker studies. Of the various quantitative proteomic analysis methods, SWATH LC–MS-based analysis has several advantages, such as reproducibility, consistency, broad coverage, and sensitivity (22, 23). Using this technique, we found that SERPINC1 and ORM1 increased more in iLN than in SLE, supporting the validity of this platform and in agreement with previous studies identifying urine ORM1 (24, 25) and ceruloplasmin (19, 24, 25) as biomarkers for active LN. Of the four ELISA-validated urine proteins, expression of ORM1 (AUC=0.886) and SERPINC1 (AUC=0.886) was significantly higher in iLN than SLE, in line with our previous study (9).

Of the 23 proteins which increased more in the iLN group than the SLE group, 19 proteins, including ORM1 and SERPINC1, were show by protein interaction network analysis to be related to the acute phase response (FDR 8E-06), cellular oxidant detoxification (FDR 8E-03) or response to stress (FDR 4E-02). The conclusions from these comparative proteomic results are that proteins involved in the acute inflammatory response are signatures of the initial stage of LN and that acute response pathway proteins such as ORM1 and SERPINC1 are potential biomarkers for monitoring the transition from SLE to initial LN without invasive kidney biopsy.

ORM, a member of the acute phase protein family, activates monocytes, induces T-cell proliferation, and promotes the secretion of pro-inflammatory cytokines, such as tumour necrosis factor α, interleukin (IL)-1, and IL-6 (12, 26). However, its biological role is poorly understood. Protein metabolomic analysis of SLE patients identified 9 proteins including ORM1 and SERPINA1 that were elevated, causing modulation of the TP53 and AMPK signalling pathways (27). Although its role in LN remains unclear, previous proteomics studies highlighted urine ORM1 as a biomarker for active LN (24, 25). Our present findings suggest that urine ORM1 is also a promising biomarker for early LN.

SERPINC1, also known as ATIII, has anti-inflammatory effects by increasing the production of prostacyclin and inhibiting thrombin-induced inflammatory cascades (10). Previous studies have identified a relationship between SERPINC1 and several kidney diseases, including nephrotic syndrome and acute kidney injury (11, 28). However, its association with LN has not been previously recognized. We provide the first evidence that it could be used as a biomarker to reflect the underlying histology in patients with LN.

Importantly, only 17.6% of patients in the iLN group had non-significant proteinuria (UPCR<500 mg/g). This and the fact that the guidelines for LN suggest renal biopsy in patients with proteinuria over UPCR 500mg/g (29, 30), indicates that we were able to identify most patients with early LN. In this regard, in the murine LN model, urine SERPINC1 and ORM1 were already significantly different from the control group at 13 weeks, at which point urine albumin was not significantly different from the control group. Therefore, the urine biomarkers identified here could be useful for early detection of LN, before, even, the development of significant proteinuria.

In addition, urine ORM1 not only identified early LN, but also was strongly associated with histologic scores for kidney pathology. There have been attempts to discover non-invasive biomarkers to reflect the renal pathology of lupus nephritis (25). In our study, ORM1 level was strongly correlated with the renal histopathology index, as was urine albumin, and it was primarily expressed in tubular membranes. The role of ORM1 in inflammatory cascades, and the fact that glomerular inflammation is a distinct pathologic hallmark of LN (31, 32), support ORM1 as a useful biomarker for early detection for LN and for the presumption of kidney damage. Since we used a lupus nephritis animal model to identify these biomarkers, further work is needed examining a longitudinal patient cohort to confirm their clinical usefulness. Lastly, other urinary abnormalities such as microscopic hematuria or cellular casts could be taken as indicative of early lupus nephritis, they were not included as variables in our analysis.

In conclusion, we have detected elevated levels of urine SERPINC1 and ORM1 in a murine model of LN, before severe proteinuria developed. Urine ORM1 levels were more closely correlated with degree of activity and chronicity index than urine albumin levels. Therefore, we suggest that urine SERPINC1 and ORM1 can be novel biomarkers for early LN.

## Data availability statement

The mass spectrometry proteomics data have been deposited to the ProteomeXchange Consortium via the PRIDE (33) partner repository with the dataset identifier PXD040099.

## Ethics statement

The studies involving humans were approved by Institutional Review Board of Asan Medical Center. The studies were conducted in accordance with the local legislation and institutional requirements. The participants provided their written informed consent to participate in this study. The animal study was approved by Institutional Review Board of Asan Medical Center. The study was conducted in accordance with the local legislation and institutional requirements.

## Author contributions

Y-GK devised the project and its main conceptual ideas. KK and JY performed the proteomics analysis. Y-EK, E-JL, DK, MJ, SH, C-KL, BY and Y-GK performed the study and wrote the manuscript. All authors contributed to the article and approved the submitted version.

## Supplementary material

Supplementary Figure 1 Opal-multiplexed immunofluorescence-stained kidney tissues in MRL/lpr and MRL/MpJ mice at 13-week. **(A)** Opal-multiplexed immunofluorescence-stained kidney tissue **(B)** Quantification of positive areas, * p<0.05

## References

[1] Anders HJ Saxena R Zhao MH Parodis I Salmon JE Mohan C. Lupus nephritis. Nat Rev Dis Primers (2020) 6(1):7. doi: 10.1038/s41572-019-0141-9 31974366

[2] Zhang L Lee G Liu X Pascoe EM Badve SV Boudville NC. Long-term outcomes of end-stage kidney disease for patients with lupus nephritis. Kidney Int (2016) 89(6):1337–45. doi: 10.1016/j.kint.2016.02.014 27165824

[3] Costenbader KH Desai A Alarcon GS Hiraki LT Shaykevich T Brookhart MA. Trends in the incidence, demographics, and outcomes of end-stage renal disease due to lupus nephritis in the US from 1995 to 2006. Arthritis Rheum (2011) 63(6):1681–8. doi: 10.1002/art.30293 PMC3106117 21445962

[4] Sexton DJ Reule S Solid C Chen SC Collins AJ Foley RN. Esrd from lupus nephritis in the United States, 1995-2010. Clin J Am Soc Nephrol (2015) 10(2):251–9. doi: 10.2215/CJN.02350314 PMC4317731 25534208

[5] Houssiau FA D'Cruz D Vianna J Hughes GR. Lupus nephritis: the significance of serological tests at the time of biopsy. Clin Exp Rheumatol (1991) 9(4):345–9. 1934681

[6] Rovin BH Zhang X. Biomarkers for lupus nephritis: the quest continues. Clin J Am Soc Nephrol (2009) 4(11):1858–65. doi: 10.2215/CJN.03530509 19729426

[7] Simard-Meilleur MC Troyanov S Roy L Dalaire E Brachemi S. Risk factors and timing of native kidney biopsy complications. Nephron Extra (2014) 4(1):42–9. doi: 10.1159/000360087 PMC4000304 24803920

[8] Malvar A Pirruccio P Alberton V Lococo B Recalde C Fazini B. Histologic versus clinical remission in proliferative lupus nephritis. Nephrol Dial Transplant (2017) 32(8):1338–44. doi: 10.1093/ndt/gfv296 PMC5837387 26250434

[9] Kwon OC Lee EJ Yeom J Hong S Lee CK Yoo B. Discovery of urine biomarkers for lupus nephritis via quantitative and comparative proteome analysis. Clin Transl Med (2021) 11(11):e638. doi: 10.1002/ctm2.638 34841703 PMC8582290

[10] Levy JH Sniecinski RM Welsby IJ Levi M. Antithrombin: anti-inflammatory properties and clinical applications. Thromb Haemost (2016) 115(4):712–28. doi: 10.1160/TH15-08-0687 26676884

[11] Lu Z Wang F Liang M. *Serpinc1/*antithrombin iii in kidney-related diseases. Clin Sci (Lond) (2017) 131(9):823–31. doi: 10.1042/CS20160669 PMC5396475 28424376

[12] Smith SA Waters NJ. Pharmacokinetic and pharmacodynamic considerations for drugs binding to alpha-1-acid glycoprotein. Pharm Res (2018) 36(2):30. doi: 10.1007/s11095-018-2551-x 30593605 PMC7089466

[13] Perry D Sang A Yin Y Zheng YY Morel L. Murine models of systemic lupus erythematosus. J BioMed Biotechnol (2011) 2011:271694. doi: 10.1155/2011/271694 21403825 PMC3042628

[14] Petri M Orbai AM Alarcon GS Gordon C Merrill JT Fortin PR. Derivation and validation of the systemic lupus international collaborating clinics classification criteria for systemic lupus erythematosus. Arthritis Rheum (2012) 64(8):2677–86. doi: 10.1002/art.34473 PMC3409311 22553077

[15] Lee JS Lee EJ Yeom J Oh JS Hong S Lee CK. Urine B-2-glycoprotein 1 as a biomarker for diagnosis of systemic lupus erythematosus. Lupus (2021) 30(8):1306–13. doi: 10.1177/09612033211014268 33966541

[16] Bajema IM Wilhelmus S Alpers CE Bruijn JA Colvin RB Cook HT. Revision of the international society of nephrology/renal pathology society classification for lupus nephritis: clarification of definitions, and modified national institutes of health activity and chronicity indices. Kidney Int (2018) 93(4):789–96. doi: 10.1016/j.kint.2017.11.023 29459092

[17] Schwartz N Michaelson JS Putterman C. Lipocalin-2, tweak, and other cytokines as urinary biomarkers for lupus nephritis. Ann N Y Acad Sci (2007) 1109:265–74. doi: 10.1196/annals.1398.032 17785315

[18] Rovin BH Birmingham DJ Nagaraja HN Yu CY Hebert LA. Biomarker discovery in human sle nephritis. Bull NYU Hosp Jt Dis (2007) 65(3):187–93. 17922668

[19] Urrego-Callejas T Álvarez SS Arias LF Reyes BO Vanegas-García AL González LA. Urinary levels of ceruloplasmin and monocyte chemoattractant protein-1 correlate with extra-capillary proliferation and chronic damage in patients with lupus nephritis. Clin Rheumatol (2021) 40(5):1853–9. doi: 10.1007/s10067-020-05454-0 33079302

[20] Urrego T Ortiz-Reyes B Vanegas-Garcia AL Munoz CH Gonzalez LA Vasquez G. Utility of urinary transferrin and ceruloplasmin in patients with systemic lupus erythematosus for differentiating patients with lupus nephritis. Reumatol Clin (Engl Ed) (2020) 16(1):17–23. doi: 10.1016/j.reuma.2018.02.002 29530762

[21] Aljaberi N Bennett M Brunner HI Devarajan P. Proteomic profiling of urine: implications for lupus nephritis. Expert Rev Proteomics (2019) 16(4):303–13. doi: 10.1080/14789450.2019.1592681 PMC6693508 30855196

[22] Anjo SI Santa C Manadas B. Swath-ms as a tool for biomarker discovery: from basic research to clinical applications. Proteomics (2017) 17(3-4):1600278. doi: 10.1002/pmic.201600278 28127880

[23] Chen MX Zhang Y Fernie AR Liu YG Zhu FY. Swath-ms-bsed poteomics: strategies and applications in plants. Trends Biotechnol (2021) 39(5):433–7. doi: 10.1016/j.tibtech.2020.09.002 33036785

[24] Suzuki M Wiers K Brooks EB Greis KD Haines K Klein-Gitelman MS. Initial validation of a novel protein biomarker panel for active pediatric lupus nephritis. Pediatr Res (2009) 65(5):530–6. doi: 10.1203/PDR.0b013e31819e4305 PMC2737257 19218887

[25] Brunner HI Bennett MR Mina R Suzuki M Petri M Kiani AN. Association of noninvasively measured renal protein biomarkers with histologic features of lupus nephritis. Arthritis Rheum (2012) 64(8):2687–97. doi: 10.1002/art.34426 PMC3381849 22328173

[26] Singh VK Fudenberg HH. Lymphocyte stimulation in vitro by orosomucoid glycoprotein. Immunol Lett (1986) 14(1):9–13. doi: 10.1016/0165-2478(86)90013-1 3492444

[27] Zeng L Chen N Liao J Shen X Song S Wang F. Metabolic analysis of potential key genes associated with systemic lupus erythematosus using liquid chromatography-mass spectrometry. Comput Math Methods Med (2021) 2021:5799348. doi: 10.1155/2021/5799348 34646335 PMC8505100

[28] Fukui H Taniguchi A Sakamoto S Kawahara S Matsunaga T Taira K. Antithrombin iii in children with various renal diseases. Pediatr Nephrol (1989) 3(2):144–8. doi: 10.1007/BF00852896 2642093

[29] Hahn BH McMahon MA Wilkinson A Wallace WD Daikh DI Fitzgerald JD. American college of rheumatology guidelines for screening, treatment, and management of lupus nephritis. Arthritis Care Res (Hoboken) (2012) 64(6):797–808. doi: 10.1002/acr.21664 22556106 PMC3437757

[30] Fanouriakis A Kostopoulou M Cheema K Anders HJ Aringer M Bajema I. 2019 Update of the joint European league against rheumatism and European renal association-European dialysis and transplant association (Eular/Era-Edta) recommendations for the management of lupus nephritis. Ann Rheum Dis (2020) 79(6):713–23. doi: 10.1136/annrheumdis-2020-216924 32220834

[31] Yu F Wu LH Tan Y Li LH Wang CL Wang WK. Tubulointerstitial lesions of patients with lupus nephritis classified by the 2003 international society of nephrology and renal pathology society system. Kidney Int (2010) 77(9):820–9. doi: 10.1038/ki.2010.13 20182417

[32] Hong S Healy H Kassianos AJ. The emerging role of renal tubular epithelial cells in the immunological pathophysiology of lupus nephritis. Front Immunol (2020) 11:578952. doi: 10.3389/fimmu.2020.578952 33072122 PMC7538705

[33] Perez-Riverol Y Bai J Bandla C Hewapathirana S García-Seisdedos D Kamatchinathan S. The PRIDE database resources in 2022: A Hub for mass spectrometry-based proteomics evidences. Nucleic Acids Res (2022) 50(D1): D543–52. 10.1093/nar/gkab1038 PMC8728295 34723319

