# Supplementary figures and images for: Urine SERPINC1/ORM1 as biomarkers for early detection of lupus nephritis in MRL-lpr mice
Source: Front Immunol. 2023 Sep 8;14:1148574. doi: 10.3389/fimmu.2023.1148574 (PMC10515280; doi:10.3389/fimmu.2023.1148574)

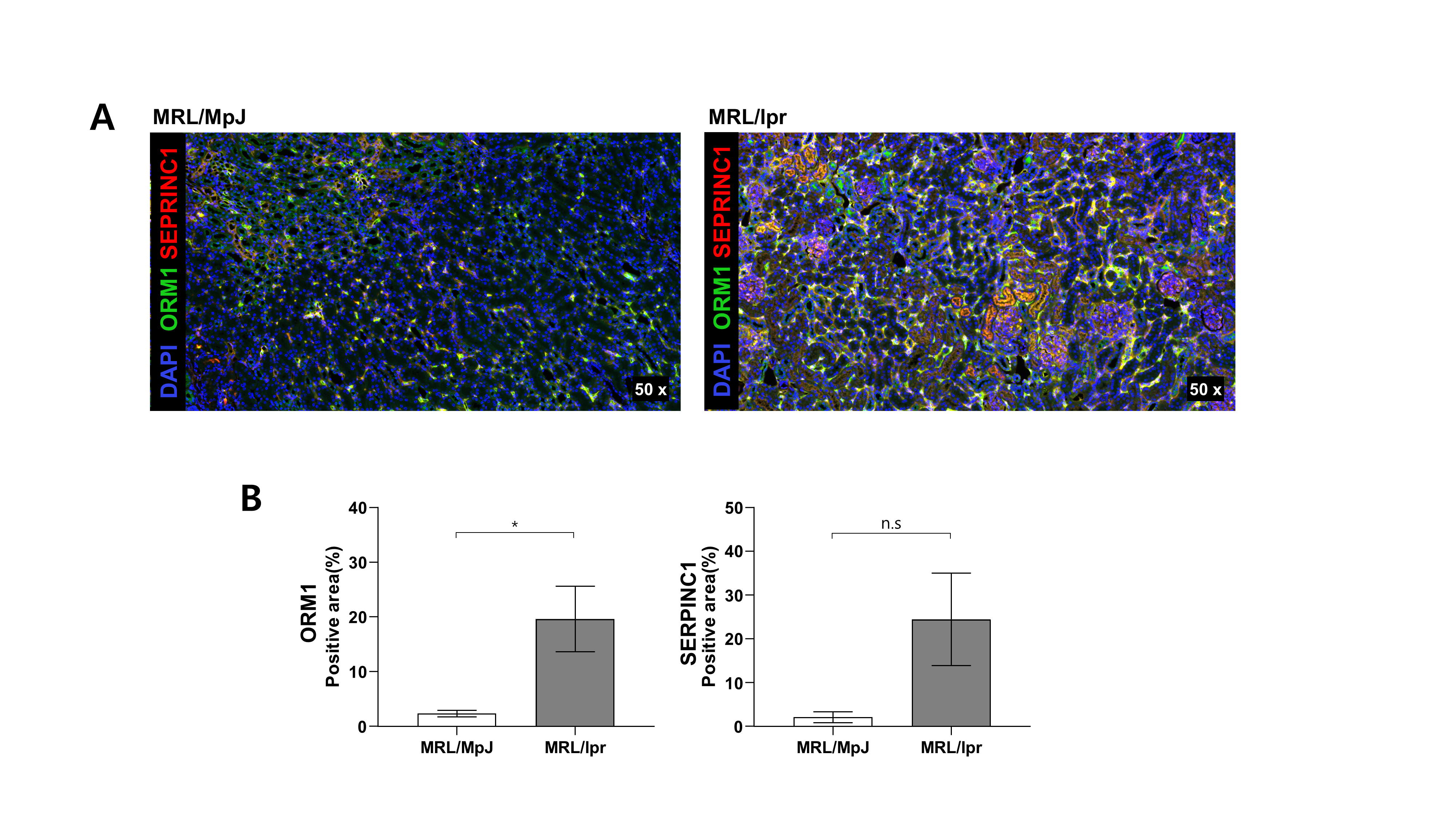

Supplement: Supplementary Figure 1 — Opal-multiplexed immunofluorescence-stained kidney tissues in MRL/lpr and MRL/MpJ mice at 13-week. (A) Opal-multiplexed immunofluorescence-stained kidney tissue (B) Quantification of positive areas, * p<0.05 [file Image_1.tif]
