# Supplementary material for: Urine SERPINC1/ORM1 as biomarkers for early detection of lupus nephritis in MRL-lpr mice
Source: Front Immunol. 2023 Sep 8;14:1148574. doi: 10.3389/fimmu.2023.1148574 (PMC10515280; doi:10.3389/fimmu.2023.1148574)
Supplement: Supplementary file 2 [file Table_1.docx]

**Supplementary Table 1. Clustering from STRING analysis**

| **#term ID** | **Term description** | **Observed  gene count** | **Background  gene count** | **Strength** | **FDR** | **Matching proteins inrRed colored cluster** |
| --- | --- | --- | --- | --- | --- | --- |
| GO:0006953 | Acute-phase response | 5 | 42 | 2.11 | 8.00E-06 | ORM1, HP, ORM2, SERPINA1, SERPINA3 |
| GO:0098869 | Cellular oxidant detoxification | 4 | 91 | 1.68 | 8.00E-03 | ALB, HBA1, HP, HBB |
| GO:0030185 | Nitric oxide transport | 2 | 5 | 2.64 | 2.00E-02 | HBA1, HBB |
| GO:0006950 | Response to stress | 11 | 3358 | 0.55 | 4.00E-02 | ORM1, ALB, LRG1, HBA1, HP, SERPINC1, TF, ORM2, SERPINA1, SERPINA3, HBB |
| GO:0043086 | Negative regulation of catalytic activity | 6 | 771 | 0.93 | 5.00E-02 | SERPINA7, SERPINA6, HP, SERPINC1, SERPINA1, SERPINA3 |
